# Mitochondria-Associated Membranes As Networking Platforms and Regulators of Cancer Cell Fate

**DOI:** 10.3389/fonc.2017.00174

**Published:** 2017-08-18

**Authors:** Maria Livia Sassano, Alexander R. van Vliet, Patrizia Agostinis

**Affiliations:** ^1^Cell Death Research and Therapy (CDRT) Laboratory, Department of Cellular and Molecular Medicine, KU Leuven University of Leuven, Leuven, Belgium

**Keywords:** endoplasmic reticulum, mitochondria, mitochondria-associated membranes, Ca^2+^ signaling, ER stress, autophagy, inflammasome, cancer cell

## Abstract

The tight cross talk between two essential organelles of the cell, the endoplasmic reticulum (ER) and mitochondria, is spatially and functionally regulated by specific microdomains known as the mitochondria-associated membranes (MAMs). MAMs are hot spots of Ca^2+^ transfer between the ER and mitochondria, and emerging data indicate their vital role in the regulation of fundamental physiological processes, chief among them mitochondria bioenergetics, proteostasis, cell death, and autophagy. Moreover, and perhaps not surprisingly, it has become clear that signaling events regulated at the ER–mitochondria intersection regulate key processes in oncogenesis and in the response of cancer cells to therapeutics. ER–mitochondria appositions have been shown to dynamically recruit oncogenes and tumor suppressors, modulating their activity and protein complex formation, adapt the bioenergetic demand of cancer cells and to regulate cell death pathways and redox signaling in cancer cells. In this review, we discuss some emerging players of the ER–mitochondria contact sites in mammalian cells, the key processes they regulate and recent evidence highlighting the role of MAMs in shaping cell-autonomous and non-autonomous signals that regulate cancer growth.

## The Endoplasmic Reticulum (ER) and Mitochondria Connection: A Brief Introduction

The interconnection between mitochondria and the endoplasmic reticulum was the first inter-organelle contact site discovered. This fascinating discovery dates back to 1959, when Copeland and Dalton identified a possible association between ER and mitochondria in cells of the pseudobranch gland of a teleost (1). Ten years later, another research group verified this discovery by using electron microscopy (2). It was only in 1990, however, after several subcellular fractionation studies (3–5), that the mitochondria-associated membranes (MAMs) were isolated as a distinct and purified structure from rat liver. Here, Vance demonstrated that MAMs work as a platform for lipid biosynthesis and transfer, by proposing an alternative non-vesicular mechanism for the inter-organelle transfer of lipid (6–8). Furthermore, eight years later, Rizzuto and co-workers uncovered another crucial functional role of the MAMs (9). They demonstrated by live cell imaging that these regions of tight contact are necessary to maintain Ca^2+^ homeostasis, suggesting the existence of Ca^2+^-enriched “hot spots,” or microdomains at the ER, which are exposed to higher concentration of Ca^2+^ than the bulk cytosol, upon IP_3_-mediated Ca^2+^ release. Further studies revealed that the ER and mitochondria interaction could occur at approximately 10–25 nm (9, 10). Importantly, at these sites of organelle interaction, the two membranes do not fuse but rather form a proteinaceous tether (11). It is now estimated that the percentage of the mitochondrial surface that appear to be physically in contact with the ER ranges between 5 and 20%, depending on the cell type (9).

Along with the recognition of the multifaceted roles of the ER and mitochondria in cellular homeostasis and cell death, these landmark discoveries sparked a growing interest in MAM biology. Consistent with this, accumulating evidence indeed indicate that disturbances in this tightly regulated interface can elicit dysfunctions in crucial cellular processes, such as apoptosis, inflammation, and autophagy, which are involved in several pathologies, including—but not limited to—cancer. Several excellent recent reviews have discussed in great details the emerging connections of MAMs with important human diseases (12–16).

However, it should be mentioned that contact sites established by the ER are not only confined to mitochondria and that through specific microdomains the ER may dynamically connect various organelles or structures in the cell. Although the complexity of this phenomenon as well as the molecular composition of these discrete ER regions are not completely solved yet, the ability of the ER to spatially and temporally coordinate interorganellar connections may have a profound physiological meaning (17). For example, it may allow mitochondria to become spatially redistributed in regions or subcellular hot spots, such as the ER–plasma membrane (PM) contacts, in order to couple extracellular cues to rapid changes in mitochondria bioenergetics and/or buffering calcium entry (18) or to the redistribution of endosomes (17). An in depth discussion of the emerging roles of membrane contact sites will not be the focus of this review and is discussed elsewhere (17).

Here, we discuss accumulating evidence demonstrating the essential role of ER–mitochondria contact sites as a molecular platform that regulates fundamental cellular processes, which are harnessed by cancer cells to support their malignant phenotype, such as sustaining ER–mitochondria Ca^2+^ signaling.

## Fundamental Structural and Functional Roles of the MAMs

Fundamental knowledge of interorganellar communication accumulated in the past decades has changed the previously accepted “mitochondria-centered” perception that the regulation of key processes, such as metabolism and cell death, could occur solely in a mitochondrion-autonomous way. Indeed, although mitochondria play a pivotal role in various aspects of cellular health, it is now appreciated that cellular processes critical for the cell’s fate, are dictated by the interactions that mitochondria dynamically establish with the ER through the MAMs. Thus, MAMs are no longer considered just a static physical bridge between ER and mitochondria but are now being increasingly considered as a crucial cellular platform for the exchange of molecular signals and protein complex formation, where critical cellular decisions in response to alterations of cellular homeostasis are taken. Several recent reviews have discussed the molecular nature of the ER–mitochondria contact sites and revealed molecular “tags,” such as palmitoylation (19), used to dynamically recruit a variety of proteins to these subdomains (14, 20, 21).

**Figure 1:**
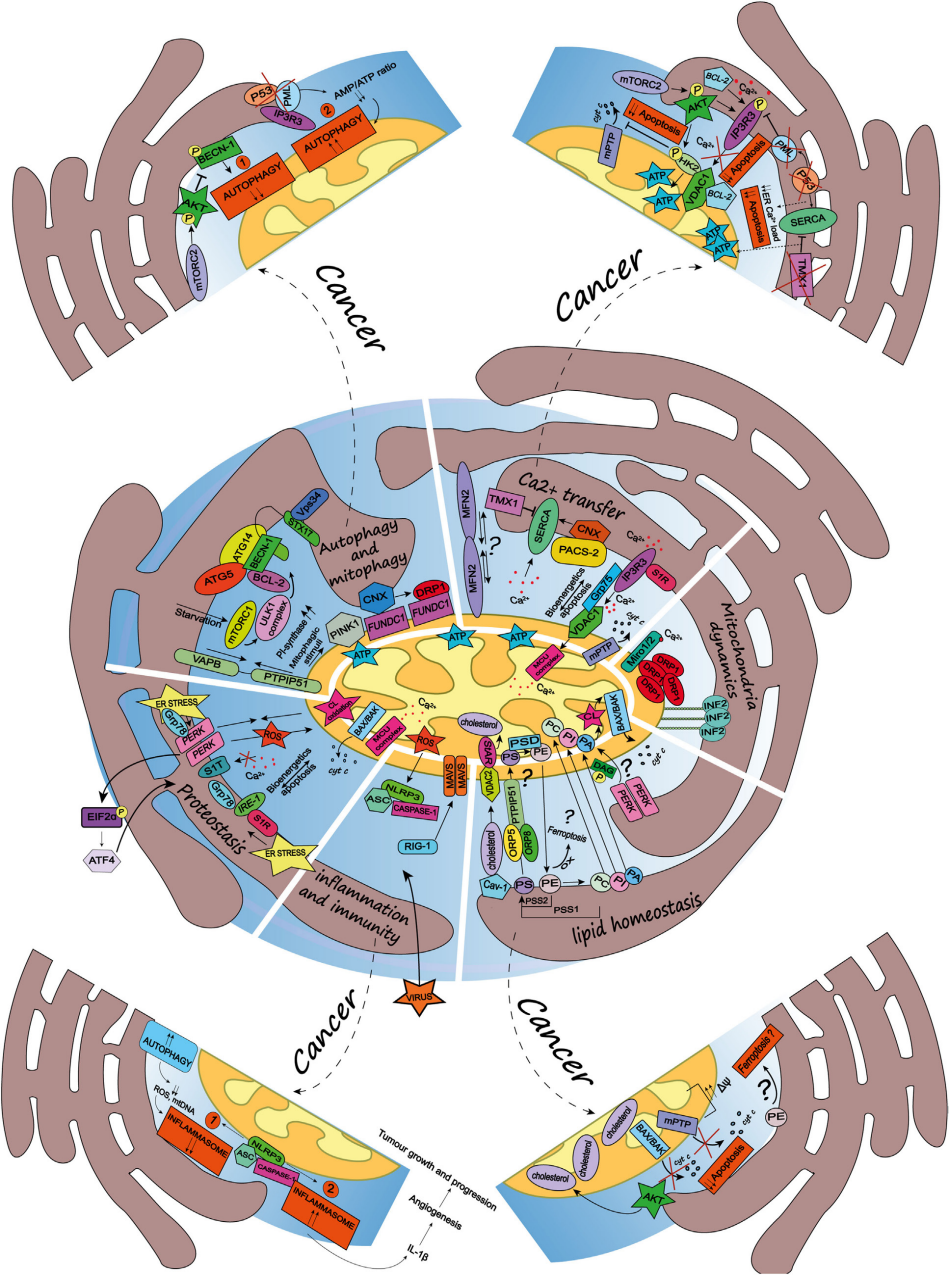
Schematic summary of the multifaceted roles of the endoplasmic reticulum (ER)-mitochondria contact sites and representation of the main pathways they regulate. Here, are represented some of the vital cellular processes regulated by the mitochondria-associated membranes (MAMs), such as autophagy and mitophagy, Ca^2+^ transfer, mitochondria dynamics, lipid homeostasis, inflammation and immunity, and proteostasis. Dashed arrows link the physiological condition of some of the MAMs-regulated cellular functions to pathways harnessed by cancer (for further details see main text). ***Autophagy and mitophagy*:** here is shown the MAMs localization of the autophagy-related gene (ATG5) and ATG14, whose recruitment is mediated by the soluble *N*-ethylmaleimide-sensitive factor attachment protein receptor (SNARE) protein syntaxin 17, upon activation of the mammalian target of rapamycin complex 1- Ulk1 unc-51 like kinase 1 (ULK1) complex, which is located in phosphatidylinositol synthase-enriched domains. Other autophagic key proteins found at MAMs are Beclin-1 and the phosphatidylinositol 3-kinase catalytic subunit type 3 (PIK3C3/Vps34). Mitophagic stimuli lead to the relocalization of the PTEN induced putative kinase 1 (PINK1) at MAMs. Additionally, the hypoxia-stimulated and mitochondria-associated FUN14 domain-containing 1, interacting with calnexin (CNX), relocates at MAMs, thus promoting Drp1 recruitment and hypoxia-induced mitophagy. Furthermore, the tethering complex vesicle-associated membrane protein-associated protein B (VAPB)–protein tyrosine phosphatase-interacting protein 51 (PTPIP51), which by tightening the ER–mitochondria contact sites, impairs rapamycin- and torin1-induced, but not starvation-induced, autophagy. ***Ca^2+^ transfer*:** here is represented the inositol 1,4,5 triphosphate receptor (IP_3_R) which is stabilized at MAMs by the chaperone Sigma1R (S1R). IP_3_R is involved in Ca^2+^ transfer from the ER storage to mitochondria by interacting with the mitochondrial voltage-dependent anion channel 1 (VDAC1), thus allowing Ca^2+^ transfer into the mitochondria through the mitochondrial calcium uniporter (MCU) and promoting mitochondrial bioenergetics and/or apoptosis, here represented by the opening of the mitochondrial permeability transition pore. Their interaction is allowed by the chaperone glucose-regulated protein 75, enriched at MAMs. Mitofusin 2 is another modulator of the interaction between ER and mitochondria (for details see the main text). The sarco/endoplasmic reticulum Ca^2+^ ATPase (SERCA) residing at the ER–mitochondria contact sites is positively modulated by chaperone CNX, whose localization at MAMs is regulated by the phosphofurin acidic cluster sorting protein 2 and negatively by the thioredoxin-related transmembrane protein 1. ***Mitochondria dynamics*:** here is represented the outer mitochondrial membrane (OMM) Rho-GTPase proteins Miro1/2 which, in presence of Ca^2+^, lose its binding with kinesin 1 (not shown), thereby reducing mitochondria motility and fostering the mitochondrial Ca^2+^ buffering. Oligomers formed by the dynamin-related guanosine triphosphatase (GTPase) dynamin-related protein 1 (Drp1) at MAMs, facilitate membrane scissions, along with actin filaments and the ER-localized protein inverted formin 2. ***Lipid homeostasis*:** the figure illustrates the lipid transfer and synthesis occurring at MAMs. The transfer of the phosphatidylserine (PS), which is formed from phosphatidylethanolamine (PE) or phosphatidylcholine (PC) by PS synthases (PSS1, PSS2), is required for the mitochondrial synthesis of PE, which is enabled by PS decarboxylase. Moreover, the ER-anchored oxysterol-binding protein-related protein (ORP) 5 and 8, are localized at MAMs where they could promote the transport of PS. PE, once exported to the ER is converted in PC. Here is also shown a speculative link to ferroptosis regulated at the MAMs, where the oxidation of polyunsaturated lipids, such as PE, occurs. The phosphatidic acid (PA) is transferred from the ER to the mitochondria as well, where it is essential for the synthesis of cardiolipin (CL), pivotal to apoptosis induction. Here is represented an interesting although speculative link involving the lipid activity kinase of the ER stress sensor RNA-dependent protein kinase (PKR)-like ER kinase (PERK), which by phosphorylating diacylglycerol can generate PA at MAMs. Cholesterol transfer into mitochondria is facilitated by the interaction with the cholesterol-binding caveolin-1, a MAM-resident protein. Moreover, the cholesterol transport steroidogenic acute regulatory protein can shuttle cholesterol from the OMM to the inner mitochondrial membrane interacting with the voltage-dependent anion channel 2. ***Inflammation and immunity*:** here is illustrated how the inflammasome is assembled at the MAMs after sensing host danger or intracellular damage, like damaged mitochondria. The inflammasome is made up of the pattern recognition receptors and nucleotide-binding domain and leucine-rich repeat-containing (NLR) protein family, NOD-like receptor family 3 and the adaptor protein apoptosis-associated speck-like protein (ASC), which recruits caspase-1 and allows its activation leading to the release of pro-inflammatory cytokines, like IL-1beta. Upon RNA virus infection, the recognition receptor RIG-I moved at MAMs, where it recruits its adaptor mitochondrial antiviral-signaling protein, thus starting an intracellular immune response. ***Proteostasis*:** here is reported that key ER stress sensors localize at MAMs, where, they can regulate signaling events linked to loss of ER homeostasis. In particular, the ER stress sensor PERK can modulate the tethering ER–mitochondria thus promoting the transfer of reactive oxygen species from the ER to the mitochondria, CL oxidation and the consequent BAX/BAK-dependent release of cytochrome *c* (cyt *c*). The PERK-activating transcription factor 4 axis of the unfolded protein response regulates the expression of S1T (truncated variants of SERCA1), thus leading to apoptosis. Moreover, here is also reported the MAMs localization of the inositol requiring enzyme 1, which interacts with S1R and Grp78, as well present at MAMs.

In the next sections, we will discuss some of the vital cellular functions and biological processes regulated by the ER–mitochondria appositions (see Figure 1).

### MAMs As Specialized Microdomains for Ca^2+^ Signaling

The importance of the ER–mitochondria platform in the regulation of Ca^2+^ homeostasis has been firmly established (22, 23). The tight tether between the ER membranes and mitochondria allows Ca^2+^ to be rapidly transferred through specialized microdomains that overcome the low apparent Ca^2+^ affinity (*K*_d_ ~ 15–20 M) of the mitochondrial Ca^2+^ uniporter (MCU) (9, 24). Many ER-associated Ca^2+^ handling proteins have been found enriched at MAMs, thus supporting the close correlation between this organellar intersection and Ca^2+^ regulation. Chiefly among these are the inositol 1,4,5 triphosphate receptors (IP_3_Rs), which together with the ryanodine receptors, represent the major ER Ca^2+^ channels (25). In particular, it has been reported that isoform 3 of the IP_3_Rs is preferentially involved in transmitting Ca^2+^ signals to mitochondria and co-localizes most strongly with them (26). The IP_3_Rs have been identified to be physically linked to the mitochondrial voltage-dependent anion channel 1 (VDAC1), located at the outer mitochondrial membrane (OMM) and associated to the MAMs as well (27). The interaction between IP_3_Rs and VDAC1 is modulated by the molecular chaperone glucose-regulated protein 75, which acts as a bridge allowing the Ca^2+^ transfer from the ER to the Ca^2+^ permeable OMM, from which the ions can easily reach the mitochondrial matrix through the mitochondrial Ca^2+^ uniporter MCU. Moreover, the sarco/endoplasmic reticulum Ca^2+^ ATPase (SERCA) pump localized to the ER membrane, is regulated by several proteins residing at the ER–mitochondria contact sites, thereby affecting Ca^2+^ flux and modulating MAMs interplay. SERCA is the ER “housekeeping” Ca^2+^ pump, which ensures the refilling of the ER Ca^2+^ storage by actively pumping this ion from the cytosol to the ER, thus restoring the Ca^2+^ balance between cytosol and ER creating a high [Ca^2+^] gradient between these regions (~0.1 μM in the cytosol and ~400 μM in the ER) (28). SERCA2b is the SERCA isoform that is particularly enriched at the ER–mitochondria contact sites (19). Among the SERCA2b interacting partners, the MAMs-resident chaperone calnexin (CNX) functions as a positive regulator while thioredoxin-related transmembrane protein 1 (TMX1) inhibits SERCA2b and thus increases mitochondrial Ca^2+^ flux (19). The phosphofurin acidic cluster sorting protein 2 (PACS-2), known to regulate the localization of CNX at the MAMs (19, 29), has also been implicated in Ca^2+^ homeostasis through its interaction with the Ca^2+^ channel transient receptor potential protein 2 (30). However, whether PACS-2 exerts this role through a direct modulation of ER–mitochondria contact sites is still a matter of debate (31).

A strong link exists between the strength of the connection between the ER and mitochondria and its Ca^2+^ trafficking role and is often used as experimental evidence in essays exploring MAMs defects. One of the major players in MAMs tethering is vesicle-associated membrane protein-associated protein B (VAPB)-protein tyrosine phosphatase-interacting protein 51 (PTPIP51) complex whose loss affects Ca^2+^ homeostasis, causing a significant delay in mitochondria Ca^2+^ uptake and, as a consequence, an increase in [Ca^2+^]_cyt_ following release from the ER store (32). A recent study indicated that the loss of both these proteins decreases the interaction between the IP_3_R3 and VDAC1, affecting the mitochondrial Ca^2+^ uptake (33). In this vein, another important ER–mitochondria tethering protein is mitofusin 2 (MFN2). Together with MFN1, MFN2 belongs to the OMM-associated fusogenic GTPases family of proteins regulating mitochondrial fusion (34). Only MFN2, however, is enriched at the ER–mitochondria contact sites and controls their stability, Ca^2+^ and lipid transfer and the morphology of both these organelles (35). In line with this, loss of MFN2 has been reported to disrupt ER–mitochondria juxtapositions and cause a decrease in the mitochondrial Ca^2+^ uptake after IP_3_-generated stimuli (35). However, this MFN2 model has been challenged recently (36). Some studies showed that MFN2 actually works antagonistically on ER–mitochondria contacts (36–38). These studies also showed that MFN2 ablation leads to an increase in ER–mitochondria coupling, thus boosting—rather than reducing—the efficiency of the IP_3_-induced Ca^2+^ transfer to mitochondria and increasing Ca^2+^ dependent cell death sensitivity (36). However, recent work has since tried to reinforce the original finding of a tethering role for MFN2 at the ER–mitochondria contact sites and its consequence for mitochondrial Ca^2+^ uptake (39). In other words, the role of MFN2 at the MAMs still remains controversial.

### MAMs As a Dynamic Bridge for Lipid Synthesis and Exchange

The ER is the major lipid factory in the cell and provides a supply of various lipids to different organelles that are unable to meet all of their own lipid requirements. The environment generated by the ER–mitochondria juxtapositions represents a privileged site for non-vesicular lipid trafficking between these organelles and facilitates transport of lipids *via* carrier proteins (40).

Although the mitochondria are capable of synthesizing several lipids through their own repertoire of enzymes, they require phosphatidylcholine (PC), phosphatidylinositol (PI), phosphatidylserine (PS), and sterols from the ER (41, 42). Mitochondria import PS through the MAMs where it is synthesized from PC or phosphatidylethanolamine (PE) by PS synthases (PSS1, PSS2). In line with this, MAMs are enriched for several phospholipid-synthesizing enzymes, such as PS and PI synthase (8, 43). After being transferred to mitochondria, PS is decarboxylated by PS decarboxylase in the inner mitochondrial membrane (IMM) to form PE. PE is then rapidly exported from the mitochondria to other organelles, such as the ER, where it is converted to PC (44). Inhibiting the enzymatic activity of PDS leads to a massive accumulation of PS in MAMs, thus supporting and confirming the involvement of the ER–mitochondria contact sites in the transfer of PS (45). The ER–mitochondria interface is also the site of phosphatidic acid (PA) transfer. PA is the precursor of cardiolipin (CL), a phospholipid enriched in the inner membrane of the mitochondria and essential for mitochondrial bioenergetics and apoptosis induction (see further section) (7, 46–48). Although PA can be made by mitochondria as well, most of the PA used for CL synthesis originates from the ER and is thus transported through the MAMs (49, 50). Interestingly, the ER stress sensor and Ser/Thr kinase PERK [RNA-dependent protein kinase (PKR)-like ER kinase], which has been shown to be enriched at the ER–mitochondria contact sites (51) is endowed with a lipid kinase and PA-generating activity (52). This raises the intriguing possibility that the lipid kinase activity of PERK may be regulated at the ER–mitochondria contact sites, a hypothesis urging experimental confirmation.

A recent study investigating the role of the ER-anchored oxysterol-binding protein-related protein (ORP) 5 and 8, which transport PS from the ER to the PM at the ER–PM contact sites, found that ORP5/8 are localized at the ER–mitochondria contact sites. MAMs localization of ORP5/8 required their interaction with the OMM protein PTPIP51 and their functional lipid transfer domain (53). Depletion of ORP5/8 caused defects in mitochondrial morphology and respiratory function, suggesting that their ability to transfer essential lipids, such as PS, within the MAMs contributes to the maintenance of mitochondrial integrity.

The lipid composition of the MAMs is critical for their functions and biological roles. MAMs are highly enriched in cholesterol, and the altering cholesterol levels itself has been shown to modulate the ER–mitochondrial connections and mitochondrial functions. Cholesterol transport into the mitochondria requires cholesterol-binding proteins. In mouse liver, the absence of the cholesterol-binding caveolin-1 (Cav-1), shown to be a MAM-resident protein, reduced the stability of ER–mitochondria contacts and increased their cholesterol content (54). Moreover, genetic ablation of Cav-1 caused a decreased electron flux through the respiratory chain, increased reactive oxygen species (ROS) generation, and apoptosis sensitization (55). Likewise, disturbed glycosphingolipid content (primarily synthesized in the ER) as caused by accumulation of GM1-ganglioside at the ER–mitochondria contact sites, in a mouse model of human lysosomal storage disease GM1-gangliosidosis, elicited stimulation of IP_3_R-mediated ER Ca^2+^ depletion, mitochondrial Ca^2+^ overload and OMM permeabilization, thus triggering ER stress-induced and mitochondria-mediated apoptosis (56) (see further section on MAMs and ER Proteostasis).

Recently, in steroid-producing cells, the cholesterol transport steroidogenic acute regulatory (StAR) protein, which shuttles cholesterol from the OMM to the IMM was found to be localized to the MAMs where it interacts with the voltage-dependent anion channel 2, enabling StAR to enter in the mitochondrial matrix and regulate steroidogenesis (57, 58). Interestingly, the generation of the intermediate StAR folding state required for the delivery of cholesterol to the mitochondria occurs at the MAMs and necessitates the presence of the ER-chaperone glucose-regulated protein 78 (GRP78) (59). This mechanism, operating at the ER–mitochondria appositions, therefore couples crucial sensors of unfolded proteins and ER proteostasis (see below), like the chaperone GRP78, to critical mitochondrial functions of steroid-producing cells (59).

Finally, the ER–mitochondria lipid connection through the MAMs could potentially modulate crucial steps in the initiation of autophagy and cell death (see next sections). A recent report shows that components of the Ulk1 unc-51 like kinase 1 (ULK1) complex involved in autophagy initiation, are recruited to ER subdomains enriched in PI synthase (60), which prime for the initiation of the autophagosome. Although not formally tested, these subdomains are reminiscent of the ER–mitochondria contact sites which have been shown to be a site for autophagosome formation (see in further section).

### MAMs As Organizers of ER and Mitochondria Trafficking and Dynamics

Both the ER and mitochondria are highly dynamic organelles, forming networks that may undergo rapid changes in the size, length, and shape, depending on metabolic and Ca^2+^ buffering needs, or in response to different cellular insults. Beside the MAMs, the mitochondria are able to establish other type of organelle-contact sites, such as the dynamic interactions with the ER and the PM, facilitating their apposition with the PM, to regulate Ca^2+^ homeostasis (61, 62).

A large body of evidence links MAMs to the regulation of intracellular transport, motility, and morphology of both the mitochondria and ER. In mammalian cells, several proteins involved in the anterograde and retrograde movement of these organelles, through kinesin 1 and cytoplasmic dynein molecular motors, respectively, are tightly regulated by local Ca^2+^ concentration rises (63). Perhaps, the most important players in this perspective are the OMM Rho-GTPase proteins Miro1/2, Ca^2+^ sensors connecting the mitochondria to kinesin 1, which have been shown to localize to the ER–mitochondria contact sites (64, 65). In subdomains that are exposed to high Ca^2+^ concentrations, such as the MAMs, Miro 1/2 lose their connection to kinesin 1. This halts mitochondrial mobility, while tightening MAMs-association, thus allowing buffering of (excessively) high cytosolic Ca^2+^ levels or stimulating oxidative phosphorylation. Beyond motility, mitochondrial fission events, driven by the dynamin-related guanosine triphosphatase (GTPase) dynamin-related protein 1 (Drp1), which forms oligomers that assemble around mitochondria to facilitate membrane scission, are orchestrated at the interface between the tubular ER and the mitochondria (66). A recent study provided compelling evidence that tubular ER is strongly associated with mitochondrial fission mechanics (67). During mitochondrial fission, the formation of constriction sites has been shown to occur at the ER–mitochondrial tether (67). Guided by the ER-localized protein inverted formin 2, actin filament polymerization in correspondence to the fission foci provides the driving force to constrict the mitochondria thus allowing Drp1 recruitment and mitochondrial fission (68). This physical intersection between tubular ER and mitochondria hinted at the involvement of the MAMs in mitochondrial division. This hypothesis has now been confirmed. An elegant study evidenced that a subset of ER–mitochondria contacts assembles the machinery for mitochondrial DNA synthesis. Thus, along with the coordination of mitochondrial motility and constriction/fission events, the ER–mitochondria contacts mark the site for mitochondrial mtDNA replication and ensure the accurate distribution of newly replicated mtDNA to daughter mitochondria upon division (69).

### MAMs, Mitochondrial Bioenergetics, and Cell Death

The rapid exchange of Ca^2+^-occurring through the MAMs is essential for mitochondrial bioenergetics and, consequently, cell fate decisions (70). The Ca^2+^-concentration in mitochondria is fundamental for several cellular processes, among these ATP production. Three dehydrogenases of the Krebs cycle are Ca^2+^-dependent enzymes, the pyruvate dehydrogenase, whose activation is due to Ca^2+^-dependent dephosphorylation, the α-ketoglutarate, the isocitrate dehydrogenases and FAD-glycerol phosphate dehydrogenase, activated directly by Ca^2+^ (71–73).

Moreover, several loss or gain-of-function studies have demonstrated that MFN2, one of the most studied modulators of the ER–mitochondria contacts, critically affects mitochondrial bioenergetics, through mechanisms that are independent of its fusogenic function (34). Thus, while MFN2 depletion reduces glucose oxidation, cellular respiration, mitochondrial membrane potential and proton leak, and mitochondrial coenzyme Q levels (74), its overexpression activates mitochondrial metabolism (34, 69). Interestingly, MFN2 also confers transient protection against starvation-induced autophagy and apoptosis by promoting mitochondrial hyperfusion and elongation (75), redox-regulated processes (76, 77) that optimize ATP production while sparing mitochondria from degradation (75).

While regulated ER-to-mitochondrial Ca^2+^ transport favors bioenergetics, it is well established that massive and prolonged mitochondrial Ca^2+^ overload triggers the opening of the mitochondrial permeability transition pore, precipitating apoptosis (72). As a result of Ca^2+^ overload and consequent OMM permeabilization, proapoptotic and caspase-activating factors, including cytochrome *c* (cyt *c*) are released in the cytoplasm (78). Once in the cytoplasm cyt *c* can also exacerbate Ca^2+^ release from IP_3_R by binding to it, thus avoiding the Ca^2+^-dependent inhibition of the receptor and amplifying caspase activation (79). To highlight the importance of ER–mitochondria juxtaposition in controlling cellular fate [for a complete overview please see Ref. (80)], work done in the lab of Hajnócsky and coworkers showed that tightening of the ER–mitochondria interaction leads to apoptotic cell death by increasing mitochondrial Ca^2+^ uptake, while loosening these links promote mitochondrial respiration and cellular survival (10, 81).

Beyond Ca^2+^ overload, MAMs could be decisive for the induction of either apoptosis or a newly described form of iron-regulated necrotic cell death, called ferroptosis, mediated by ROS and hallmarked by the accumulation of lipid peroxidation products (51, 82). During apoptosis induced by photo-oxidative ER stress (i.e., hypericin-mediated photodynamic therapy (83)), weakening of the ER–mitochondria contacts by the depletion of PERK protected cancer cells from rapid ROS-induced CL oxidation—preceding the unfolded protein response (UPR)-mediated cell death pathway—and the consequent release of cyt *c* in the cytosol (51). Indeed, during apoptosis, the interaction of CL with cyt *c* in the presence of mitochondrial ROS, drives the formation of cyt *c*/cardiolipin peroxidase complexes that oxidize CL and cause the release of cyt *c* from mitochondria into the cytosol (48, 84).

But could MAMs also be relevant for the induction of ferropotosis? Recent studies using quantitative redox lipidomics showed that oxidation of polyunsaturated lipids, the molecular executioners of ferroptosis, occurs in ER subdomains and involves oxidation of one class of phospholipids; PE molecules with arachidonoyl (AA) or adrenoyl (AdA) fatty acyls, driven by lipoxygenase (15-LOX) (85). A direct link between ferroptosis and MAMs has not been explored yet. Notably, acyl-CoA synthetase long-chain family member 4 (ACSL4 or FACL4), which catalyzes the synthesis of AA thus promoting its esterification in phospholipids and conferring ferroptosis susceptibility (86), is found enriched at the MAMs (87). Thus, these observations raise the tantalizing possibility that ROS-mediated death signals generated at the ER–mitochondria contact sites could be decisive to trigger either apoptosis of ferroptosis, depending on the type of oxidized phospholipids.

### MAMs and ER Proteostasis

Accumulating evidence indicates the tight connection between the UPR and MAMs components. The UPR is a conserved intracellular pathway engaged by various physiological and stressful cues triggering the accumulation of unfolded proteins in the ER, a condition known as ER stress [extensively reviewed in Ref. (88, 89)]. ER–mitochondria contacts establish an intense and mutual cross talk that facilitates stress responses evoked by the UPR (14, 90, 91). Perhaps, the strongest evidence in support of this link is the finding that key ER stress sensors have been shown to regulate signaling events linked to loss of ER homeostasis by directly localizing at the MAMs and/or by regulating the expression of ER–mitochondria tethers. In particular, our lab discovered that the ER stress sensor, PERK is an integral member of the MAMs and has a tethering role at the ER-mitochondria contact sites (51). PERK-deficient cells display weakened ER–mitochondria contact sites and increased apoptosis resistance against agents that simultaneously mobilize Ca^2+^ and induce ER stress through ROS (51). Interestingly, one study showed that MFN2 interacts with PERK and prevents its activation (92). In response to ER stress, PERK ablation increases apoptosis in MFN2-ablated cells, but it also partially rescues the aberrant mitochondrial Ca^2+^ content and the fragmentation of the mitochondrial network, caused by loss of MFN2 (92). While this study would suggest that MFN2 deficiency causes mitochondrial aberrations through sustained activation of the PERK pathway of the UPR, the still controversial aspects around the role of MFN2 in ER–mitochondria tethering and the presence of PERK at the MAMs, ask for further mechanistic analysis of this signaling cross talk.

Moreover, the PERK-activating transcription factor 4 (ATF4) axis of the UPR was shown to be required for the induction of S1T, a truncated variants of SERCA1 which localized to the MAMs and led to an increasing number of contact sites, mitochondrial Ca^2+^ overload and an inhibition of mitochondrial movement, which ultimately triggered apoptosis (93). This suggests that following ER stress, the activation of the PERK-ATF4 pathway may induce a transcriptional program that reinforces ER–mitochondria contact sites. In line with this, the PERK-ATF4 pathway can also lead to the expression of E3 ubiquitin ligase Parkin (94), which has been reported to increase ER–mitochondrial coupling and Ca^2+^ transfer favoring mitochondrial bioenergetics (95). This highlights that activation of the PERK-ATF4 pathway of the UPR, may contextually regulate cellular fate through the upregulation of MAMs-resident proteins. Other members of the UPR, such as the inositol requiring enzyme 1 (IRE1), appear to localize to or have a signaling role at the ER–mitochondria contact sites during ER stress. Interestingly, during ER stress, IRE1 dimerization is facilitated through the interaction with the MAMs-resident ER-chaperone sigma1 receptor (96), which operates as a Ca^2+^ sensor and dissociates from GRP78 upon ER Ca^2+^ depletion to favor IP_3_R-mediated Ca^2+^ transfer to mitochondria (97). Although the exact role of IRE1 at the MAMs still needs to be fully defined, the co-presence of key sensors of the UPR and GRP78 (a chaperone involved in their activation) with Ca^2+^ sensors and channels at the ER–mitochondria contact sites, suggests that MAMs are a critical signaling hub coordinating responses to changes in ER Ca^2+^ homeostasis.

The mitochondrial contact sites are not the only site of close contact that the ER is able to form with organelles in order to perform its various functions and maintain the ER homeostasis. The ER–PM juxtapositions are one of the most important ER–organellar contact sites, together with MAMs, which are responsible for orchestrating several cellular responses including—but not limited to—Ca^2+^ homeostasis, lipid-mediated signaling, metabolism, cell death, and exocytosis (98). In particular, ER–PM juxtapositions are enabled by morphological changes in the ER and the expansion of thin ER structures, mainly known as cortical ER, toward the PM, thus allowing the ER to rapidly sense and respond to alterations in ER homeostasis by modifying the composition of proteins and lipid membranes. Recently we revealed that PERK, beyond its ability to maintain the ER–mitochondria appositions (51), enables rapid and efficient formation of ER–PM contact sites upon ER Ca^2+^ store depletion, largely through a UPR independent mechanism (99). PERK, through its interaction with Filamin A (FLNA), a major F-actin binding protein, and remodeling of the F-actin network allows the expansion of the ER tubular network underneath the PM and the recruitment of the ER–PM tethers Stromal interaction molecule 1 (STIM1) and Extended-Synaptogamin 1 (E-Syt1) at the PM in response to agents eliciting ER Ca^2+^ depletion and store operated Ca^2+^ entry (99).

Thus, it is increasingly becoming clear that key ER stress sensors like PERK orchestrate the communication between the ER and other crucial compartments/organelles, like the PM, mitochondria and nucleus, by regulating ER homeostasis through membrane contact sites as well as UPR-mediated transcriptional regulation.

### MAMs and Autophagy

Endoplasmic reticulum–mitochondria contact sites have been linked to autophagy initiation. Autophagy is a tightly regulated lysosomal pathway for the recycling of cytoplasmic material, including proteins and organelles, which serves as a vital quality control mechanism to preserve cellular integrity and homeostasis and as an alternative energy source to survive in nutrient-deprived conditions [for a detailed overview of the molecular pathways regulating autophagy see Ref. (100, 101)]. Interestingly, although the origin of the autophagosomal membrane still remains a matter of debate it has recently been demonstrated that autophagosomes form at the ER–mitochondria contact sites, thus assuming that this interplay could play a critical role in autophagy (102, 103). Recently, it has been reported that the autophagosome-formation marker ATG5 and the pre-autophagosome marker ATG14, whose recruitment is regulated by soluble *N*-ethylmaleimide-sensitive factor attachment protein receptor (SNARE) protein syntaxin 17 (STX17), move and localize at MAMs (103). Accordingly, other key proteins involved in autophagosome formation have been found recruited at MAMs under starvation, chief among these are the pro-autophagic proteins Beclin-1 (BECN1) and the phosphatidylinositol 3-kinase catalytic subunit type 3 (PIK3C3/Vps34) (103). Additionally, the lipid raft-composition of the MAMs could facilitate initial organelle interaction, which leads to the formation of autophagosomes, since these lipid microdomains have been recently identified as important actors of the autophagic process (104).

Relocalization of key proteins of the mitophagy machinery such as the phosphatase and tensin homolog deleted on chromosome 10 (PTEN) induced putative kinase 1 (PINK1), a mediator of Parkin-dependent mitophagy, to the MAMs has been shown to occur in response to pro-mitophagic stimuli, indicating that the ER–mitochondria contact sites are a privileged site to tag mitochondria for clearance (105). Strengthening the connection with the mitophagy machinery, the hypoxia-stimulated and mitochondria-associated FUN14 domain-containing 1 (FUNDC1), has been found to serve as an adaptor for Drp1 recruitment at the ER–mitochondria interface, thereby inducing hypoxia-mediated mitochondrial fission and mitophagy (106).

However, a new study shows that the tightening of the ER–mitochondria tether inhibits rather than promotes autophagy (14, 33). Although it seems contradictory, this elegant study sheds light on the mechanism regulating autophagy by MAMs, suggesting that the ER–mitochondria contact sites could play different roles in autophagy signaling depending on the stimuli. In fact, the overexpression of the tethering complex VAPB-PTPIP51, tightening the ER–mitochondria coupling, impairs rapamycin- and torin1-induced, but not starvation-induced, autophagy (33). Additionally, the mechanism by which the VAPB-PTPIP51 complex regulates autophagy is due to their ability in modulating the ER–mitochondria Ca^2+^ signaling, further supporting the role of MAMs as the crucial signaling hub of autophagy regulation.

### MAMs As a Molecular Platform for the Inflammasome and Mitochondrial Antiviral-Signaling Protein (MAVS)

Given the tight link, as mentioned before, between ER stress and MAMs, it is perhaps not unexpected that ER–mitochondria juxtapositions are emerging as regulators of inflammation and immunity. ER–mitochondria contact sites have been shown to modulate the activity of a member of the pattern recognition receptors (PRRs) and nucleotide-binding domain and leucine-rich repeat-containing (NLR) protein family, NOD-like receptor family 3 (NLRP3). NLR proteins are involved in sensing host “dangers” elicited by foreign pathogens, tissue and intracellular damage, such as molecules released by damaged mitochondria, and in mounting an inflammatory response (107). This response is driven by the assembly of an inflammasome, a multimolecular complex composed of an NLR and the adaptor protein apoptosis-associated speck-like protein (ASC), which recruits caspase-1. Caspase-1 activation in the complex leads to the proteolytic maturation of the pro-inflammatory cytokine interleukin 1β (IL-1β) and interleukin 18 (IL-18) (108). Recently, it has been shown that upon detection of mitochondrial ROS, and release of mitochondrial factors, NLRP3 relocates from the ER to the ER–mitochondria appositions, thus sensing the mitochondrial alteration and assembling the inflammasome (109). Interestingly, upon RNA virus infection, the cytosolic pathogen recognition receptor RIG-I is recruited at the ER–mitochondria contact sites and recruits its adaptor mitochondrial antiviral-signalling protein (MAVS), to drive an intracellular immune synapse that directs antiviral innate immunity (110).

These observations indicate that ER–mitochondria contacts sites can modulate important cell-non-autonomous processes, like inflammation and immunity, that are emerging as key modulators of the tumor microenvironment and therapeutic responses (111).

## Functional Alterations of the ER–Mitochondria Contact Sites in Cancer Cells

Given the crucial role of MAMs in cellular homeostasis and cellular fate, it is not surprising that an increasing number of reports have highlighted a close intersection between processes that are altered during carcinogenesis and ER–mitochondria contact sites. In particular, it is intriguing to note that several oncogenes and tumor suppressors have been found to target dynamically these ER microdomains. In line with this, it is increasingly becoming clear that signaling events regulated at the MAMs modulate cancer cell’s capability to adapt to intracellular stress, caused by oncogenic perturbations, thereby decreasing the cellular responsiveness to cell death signaling, as well as to extracellular cues found in the tumor microenvironment. Beyond the expression of oncogenes or loss-of-function of tumor suppressors, cancer cells hijack key homeostatic processes, which, as mentioned before are highly regulated at the intersection between the ER and mitochondria. For example, perturbations of Ca^2+^-signaling, autophagy and the inflammasome, may favor both cancer cell-autonomous and extrinsic features that enable cancer progression and therapy responses. Here, we discuss some of the emerging traits of the cancer cells that have been linked, or can be associated, to intracellular pathways and processes regulated at the ER–mitochondria appositions (see Figure 1).

### MAMs As Modulators of the Mitochondrial Lipid Composition of Cancer Cells

It has been reported that the lipid composition of the IMM is altered in cancer cells (112, 113). In particular, several types of tumors show an increase in the content of cholesterol (114, 115) and phospholipids with shorter and less unsaturated acyl chains (116, 117). The mitochondrial inner membrane lipid composition could affect mitochondrial bioenergetics and impact on apoptotic cell death, altering the susceptibility to apoptotic stimuli (113, 114). For example, higher cholesterol content decreases the permeability of the IMM, thus affecting the passive transfer of protons and increasing the Δψ, effects that result in an impaired respiratory chain activity (118). Moreover, increased cholesterol is commonly associated in several solid tumors to the overactivation of the Akt pathway (119, 120). This observation has been linked to an impairment of B-cell lymphoma 2 (Bcl-2)-Associated X Protein (Bax)-driven OMM and cyt *c* release (115). As discussed above, the lipid composition of the MAMs could modulate different, not overlapping types of cell death, including apoptosis and ferroptosis, a form of regulated necrosis driven by lipid peroxides (121). This may offer an interesting therapeutic possibility given that alternative cell death pathways may be exploited to break down apoptosis resistance in cancer cells, and suggests that lipid manipulations of MAMs could be targeted to favor the cytotoxic activity of ferroptosis inducers.

### MAMs As a Lair of Proto-Oncogenes and Tumor Suppressor

Emerging cellular and *in vivo* evidence indicate that perturbation of Ca^2+^ homeostasis is one of the vital mechanism through which oncogenes and tumor suppressors impact cancer cell fate (81). Accordingly, and as discussed below, oncogenes and tumor suppressors engage in complex interactions at the ER–mitochondria contact sites to directly shape Ca^2+^ flux from the main cellular storage, the ER, to the cellular energy–supplier, the mitochondria.

### The IP_3_Rs As a Molecular Scaffold for the Action of Oncogenes and Tumor Suppressors

As mentioned above the IP_3_Rs are the most important Ca^2+^-transport systems involved in maintaining Ca^2+^ homeostasis between ER and mitochondria. Given that these channels are directly responsible for mitochondrial Ca^2+^ uptake, and considering the role of Ca^2+^ signaling in cancer cells (122), IP_3_R activity has been shown to be directly or indirectly regulated by several oncogenes. It is now firmly established that the functionality of the IP_3_Rs is modulated by post-transcriptional modifications, including phosphorylation. Several oncogenes and tumor suppressors have been shown to act by modulating IP_3_R phosphorylation state in order to modify the rate of Ca^2+^ efflux from intracellular stores and, as a consequence, altering the cellular response to apoptosis (123, 124). The proto-oncogene serine/threonine kinase Akt (also known as protein kinase B), is able to phosphorylate all the isoforms of the IP_3_R in a conserved consensus sequence found in the cytosol-exposed C-terminal tail (125, 126). Accordingly, in cancer cells in which Akt is upregulated, IP_3_Rs are hyper-phosphorylated (126) and as a result, Ca^2+^ efflux from ER to mitochondria is blunted, thus protecting cells from apoptotic stimuli (127, 128). Interestingly, Akt mainly phosphorylates the isoform 3 IP_3_R (IP_3_R3), which is enriched at the ER–mitochondria interface, thus suggesting that this antiapoptotic function of Akt requires its compartmentalization at the MAMs (128). Interestingly, the mechanistic target of the Rapamycin complex 2 (mTORc2), which activates Akt, is also found at the ER–mitochondria appositions (129). Along with regulation of the phosphorylation state of IP_3_R and Ca^2+^ flux, the mTORc2/Akt complex controls mitochondrial metabolism and physiology, through the phosphorylation of the glycolytic enzyme hexokinase 2, thus promoting cancer cell’s aerobic glycolysis (Warburg effect) and preventing mitochondrial apoptosis (130, 131).

On the other side of the coin, the phosphorylation state of IP_3_R3 is, directly or indirectly, modulated by the recruitment of different tumor suppressors, including the lipid phosphatase and negative regulator of Akt, the PTEN (132, 133), p53 (134) and the promyelocytic leukemia (PML) protein (135). The fraction of PML localized at the MAMs (135) is essential to maintain a normal Ca^2+^ flux between ER and mitochondria since the absence of PML leads to a decrease in the amplitude of Ca^2+^ signals after induction of IP_3_-mediated Ca^2+^ release. At the MAMs, PML resides in a multi-protein complex, consisting of IP_3_R3, Akt and the protein phosphatase 2A (PP2A). PML appeared to be vital for the binding to IP_3_R of PP2A, which, by de-phosphorylating Akt, inhibits its activation (135, 136). The consequent reduced IP_3_R3 phosphorylation, ultimately drives Ca^2+^ flux from ER to mitochondria, thus preserving cellular susceptibility to Ca^2+^-dependent apoptotic signals. In addition, recent data showed that PML recruitment at the ER–mitochondria contact sites is modulated by the tumor suppressor p53 (134), thus suggesting that key tumor suppressors cooperate to hinder oncogenic signaling, by physically localizing to this strategic subcellular compartment.

### The SERCA Pump As a Target of Oncogenes and Tumor Suppressors

The modulation of the SERCA pump functional activity is another common mechanism through which MAMs-located oncogenes and tumor suppressors, by remodeling the ER-Ca^2+^ steady state, can modulate cancer cell fate (81, 137, 138). For example, the non-nuclear proapoptotic function of the tumor suppressor p53 at MAMs involves its interaction with the SERCA pumps (139). Recent work showed that the pool of MAMs-associated p53 keeps the oxidation state of SERCA in check, thereby favoring its activity and filling of the ER Ca^2+^ store. Upon an apoptotic stress or treatment with an anticancer agent, this p53 mediated mechanism at the ER–mitochondria interface allows rapid mitochondrial Ca^2+^ overload, thus precipitating apoptotic cell death (139). Opposite to this, the redox-sensitive oxidoreductase TMX1 binds and inhibits the SERCA2b pump at the ER–mitochondria appositions, under oxidizing conditions, thus decreasing ER Ca^2+^ load and impairing mitochondrial respiration (19). This is in line with the notion that low-level constitutive IP_3_-mediated Ca^2+^ release supports oxidative phosphorylation of cancer cells, in the absence of which low ATP levels may trigger autophagy (70). Accordingly, cancer cells displaying lower levels of TMX1 show an increased ER Ca^2+^ content and an accelerated cytosolic Ca^2+^ clearance, along with a reduced ability of the ER to direct Ca^2+^ toward mitochondria. Interestingly, human cancer cells with impaired expression of TMX1 levels, when xenografted in nude mice exhibited faster tumor growth, which was explained by a metabolic reprogramming and induction of a Warburg phenotype in these cancer cells (19).

### Interaction of Antiapoptotic Bcl-2 Family Members with Components of the MAMs

Beyond Akt and various antiapoptotic Bcl-2 family members including Bcl-2 and B-cell lymphoma-extra large (Bcl-XL), which are commonly overexpressed in a variety of cancers (140), have been proposed to suppress the IP_3_-mediated Ca^2+^-flux from ER to mitochondria by targeting the IP_3_Rs at the ER-mitochondria contact sites (141, 142). Although a full understanding of the antiapoptotic function of the Bcl-2 oncogene at the ER–mitochondria contact site is still elusive, Bcl-2 is thought to either lower the ER Ca^2+^-store content, or act directly on Ca^2+^-discharge from ER to the mitochondria [extensively reviewed in Ref. (143)]. Recently, the specific BH4 domain of Bcl-2 was shown to directly target all the three isoforms of IP_3_Rs and to inhibit their activity, hence suppressing ER Ca^2+^ release and reducing the cellular sensitivity to apoptosis (141, 144, 145). Likewise, the binding between Bcl-XL the IP_3_Rs has recently been determined to be fundamental for exerting its antiapoptotic functions (146–148). Both the antiapoptotic proteins Bcl-2 and Bcl-XL inhibit the MAMs-resident voltage-dependent anion channel VDAC1, by controlling its permeability to Ca^2+^ and thereby preventing cyt *c* release and apoptotic cell death (149–152). Although both Bcl-2 and Bcl-XL directly bind to the N-terminal region of VDAC1 (153, 154), the regions involved in this binding are different. In fact, whereas the BH4 domain of Bcl-XL is able to selectively target and inhibit the N-terminal domain of VDAC1, the BH4 domain of Bcl-2 is uniquely involved in inhibiting the IP_3_Rs (151). However, the functional role of the Bcl-XL-VDAC1 is still a matter of debate since a different impact of Bcl-XL interaction on VDAC1’s proprieties has been reported (155).

### MAMs As Regulators of Autophagy in Cancer

Emerging evidence indicates that autophagy has a highly dynamic role during tumorigenesis and can act either as a tumor-promoting or as a tumor-suppressing process, depending on the different stages of the tumor and the tight cross talk between cancer cells and stromal cells in the tumor microenvironment. Mechanistically, the tumor suppressor role of autophagy has been ascribed to the vital cell-autonomous functions of autophagy in maintaining cellular integrity and mitigating cellular damage under conditions of metabolic stress, including (but not limited to) the removal of damaged mitochondria and ROS [reviewed in Ref. (156)]. On the other hand, during tumor development, heightened autophagy becomes vitally required to maintain cancer cell’s elevated energy balance, through the recycling of intracellular components into biosynthetic pathways or ATP synthesis, and to regulate secretion of pro-tumorigenic factors. Thus in established tumors, autophagy allows cancer cells to plastically adapt to the increasingly hostile tumor microenvironment, characterized by poor nutrient availability, and foster tumor growth by establishing an intense interface with stromal cells (157, 158).

As mentioned before, several oncogenes and tumor suppressors that have been found localized at MAMs are also critical modulators of autophagy (159–162). In particular, the MAMs-associated mTORc2/Akt complex could inhibit autophagy by phosphorylating and inhibiting BECN1, an interacting partner of IP_3_R3 and Bcl-2 (162–164). Furthermore, autophagy induction in cancer cells is under the control of MAMs-associated p53 and PML. The absence of p53 or mislocalization of PML away from MAMs activates autophagy in response to cellular stress, suggesting that p53 via PML suppresses cytoprotective autophagy (134). These data indicate that the p53-PML interaction at the ER-mitochondria appositions, by regulating the transfer of Ca^2+^ from the ER to the mitochondria, on one hand, favors Ca^2+^-dependent apoptosis, while on the other, suppresses autophagy. Indeed the loss of PML, by decreasing constitutive Ca^2+^-release reduces mitochondrial respiration and ATP production, which results in the stimulation of autophagy *via* AMPK activation. This may explain why the loss of PML from MAMs promoted tumor growth and increased chemoresistance by simultaneously increasing resistance to apoptotic stimuli and, through the stimulation of autophagy, adaptation to metabolic stress and anticancer therapy-mediated cellular damage (134). Interestingly, in cancer cells with constitutively low level of ER-to-mitochondrial Ca^2+^ transfer, AMPK-dependent autophagy activation is not sufficient to maintain cancer cell survival. Inhibition of IP_3_R attenuates the growth of B16 melanomas, implanted in immunocompromised mice, due to a severe bioenergetics failure that triggers necrotic cancer cell death (165). This study further unravels a cancer cell vulnerability, which is imparted by the dependency on constitutive Ca^2+^ transfer to mitochondria for viability, at least in cancer cells still relying on mitochondrial respiration to supply their energy needs. It moreover suggests that targeting the ER–mitochondria interface, and breaking down the “mitochondrial Ca^2+^ addiction,” may provide a novel therapeutic strategy to halt tumor growth. A hypothesis that should be further tested in immunocompetent mice, to assess the impact of inhibition of cancer cell-associated ER–mitochondria Ca^2+^ transfer and perturbations of ER–mitochondria appositions, on a fully competent tumor microenvironment.

### MAMs As Regulators of Cancer Cell-Non-Autonomous Functions

As mentioned above, recent research has highlighted an important role for MAMs in the regulation of the NLRP3 inflammasome. The role of the inflammasomes is best appreciated in innate immune cells, such as myeloid cells, but certain aggressive cancer cells, such as melanoma cells, are able to produce autonomously high levels of pro-inflammatory cytokines, such as IL-1 β through the activation of the inflammasome (166). In tumors, activation of the inflammasome has contextual pro-tumorigenic and anti-tumorigenic roles, reflecting the double-edged role played by inflammation in cancer progression (167, 168). Indeed, the main cytokines produced by the assembly of the NLRP3 inflammasome, IL-1β and IL-18, exert pleiotropic effects in inflammation, immunosurveillance and therapy responses and have both pro- and anti-tumorigenic functions depending on the context and type of cancer, as well as in response to anticancer therapies and their ability to induce immunogenic cell death (169, 170). Interestingly, MAMs could have a central role in priming the activation of the inflammasome and at the same time preventing excessive inflammation through the recruitment of the autophagy machinery. In line with this, by the removal of aged and malfunctioning mitochondria, autophagy has been shown to downregulate the activation of the NLRP3 inflammasome at the ER-mitochondria contact sites, thereby preventing the release of crucial NLRP3 activators, including mtDNA and mitochondrial ROS (171). Thus although speculative, this connection deserves to be further tested in the near future.

Finally, a recent study proposes an intriguing link between ER-mitochondria contacts sites and immunosurveillance (172). In glioblastoma, a highly aggressive and heterogeneous tumor, glioma stem-like cells (GSCs) displayed an increased susceptibility to killing by cytotoxic T lymphocytes and natural killer cells—the two major players of the immunosurveillance process—than their differentiated glioma cells (GDCs). This demonstrated increased susceptibility of GSCs to T cell killing both *in vitro* and *in vivo*. This also correlated to the reduced presence of surface sialylated glycans, which in turn extended the duration of immune synapses formed with cytotoxic T cells when compared to GDCs. Remarkably, GSCs had more fragmented mitochondria compared to GDCs and reduced ER-mitochondria tethering along with reduced expression of Mfn2. Importantly, the causative link between reduced MAMs and low levels of sialylated glycans in GSCs was evidenced by artificially forcing ER–mitochondria tethering, which resulted in increased cell surface glycans and reduced killing by CTLs (172). Although it is still elusive how mitochondrial morphology and ER–mitochondria appositions regulate the composition and expression of surface glycans, this study provides the first evidence that MAMs can modify the cell surface glycoprotein glycocalyx, a key determinant of immune modulation harnessed by cancer cells to evade control from both innate and adaptive immune destruction (173).

## Conclusion

The increasingly important and pleiotropic role of ER–mitochondria tethers in the coordination of key cellular functions has been elucidated by the growing interest in this fundamental cell biology process sparked in the last decades. Along with the complex and dynamic molecular composition of the MAMs, several studies have highlighted the fundamental signaling role of these ER subdomains and their relevance for cancer biology and treatment. However, several outstanding questions still need to be answered before reaching a complete mechanistic and functional understanding of the ER–mitochondria interface. For example, how do MAMs regulate the strength and outcome of signaling pathways, such as those engaged by the loss of ER homeostasis, which are crucial for cancer cell survival, secretion, inflammation, and therapy responses? Which are the key mediators of this process and can they be harnessed for therapeutic intervention in cancer therapy? Given their role in lipid trafficking between the ER and mitochondria, do MAMs strategically control the type of cancer cell death induced by metabolic alterations or cellular stress? How do ER–mitochondria contact sites regulate immunosurveillance mechanisms through changes in the surface composition of the cancer cells and what is the role of MAMs in the release of mitochondria-derived danger signals, such as mtDNA and formyl peptides?

In this context, the use of modulators of ER–mitochondria tethers, such as the recently developed MFN2 inhibitors (174), could provide a relevant approach to further study cancer cell-intrinsic (such as the ER-to-mitochondria Ca^2+^ dependence for survival) and extrinsic (immunosurveillance mechanisms, inflammation and release of mitochondria-derived danger signals) processes that are modulated by ER–mitochondria contact sites. This further urges clarity about the role of MFN2 in ER–mitochondria appositions in both normal and cancerous cells. Finally, it is still not clear whether ER–mitochondria contact sites modulate the mitochondrial UPR, an emerging process modulating mitochondrial proteostasis (175). All these are outstanding questions that await future studies.

## Author Contributions

MS and PA conceived and wrote the manuscript. AV helped in improving and editing the manuscript. PA supervised and critically revised the manuscript.

## Conflict of Interest Statement

The authors declare that the research was conducted in the absence of any commercial or financial relationships that could be construed as a potential conflict of interest.

## Abbreviations

AA, arachidonoyl; ATF4, activating transcription factor 4; ATG, autophagy-related gene; Bcl-2, B-cell lymphoma 2; Bcl-XL, B-cell lymphoma-extra large; BECN1, Beclin-1; Ca^2 +^, calcium; Cav-1, caveolin-1; CL, cardiolipin; CNX, calnexin; cyt *c*, cytochrome *c*; Drp1, dynamin-related protein 1; ER, endoplasmic reticulum; Grp78, glucose-regulated protein 78; HK2, hexokinase 2; IL-1β, interleukin 1β; IL-18, interleukin 18; IP_3_, inositol trisphosphate; IP_3_R, inositol 1, 4, 5-trisphosphate receptor; IP_3_R3, inositol 1, 4, 5-trisphosphate receptor 3; IRE1, inositol requiring enzyme 1; MAMs, mitochondria-associated membranes; MAVS, mitochondrial antiviral-signaling protein; MCU, mitochondrial calcium uniporter; MFN2, mitofusin 2; mTORC2, mammalian target of rapamycin 2; NLRP3, NOD-like receptor family 3; OMM, outer mitochondrial membrane; ORP5, oxysterol-binding protein (OSBP)–related protein (ORP) 5; ORP8, oxysterol-binding protein (OSBP)–related protein (ORP) 8; PA, phosphatidic acid; PACS-2, phosphofurin acidic cluster sorting protein 2; PC, phosphatidylcholine; PE, phosphatidylethanolamine; PERK, double stranded RNA-activated protein kinase (PKR)-like ER kinase; PI, phosphatidylinositol; PM, plasma membrane; PML, promyelocytic leukemia; PP2A, protein phosphatase 2A; PS, phosphatidylserine; PTEN, phosphatase and tensin homolog deleted on chromosome 10; PTPIP51, protein tyrosine phosphatase-interacting protein 51; ROS, reactive oxygen species; SERCA, sarco/endoplasmic reticulum Ca^2+^ ATPase; SERCA2b, sarco/endoplasmic reticulum Ca^2 +^ ATPase isoform 2b; Sigma1 receptor, S1R; StAR, cholesterol transport steroidogenic acute regulatory protein; TMX, thioredoxin-related transmembrane protein; VAPB, vesicle-associated membrane protein-associated protein B; VDAC1, voltage-dependent anion channel 1; VDAC2, voltage-dependent anion channel 2.
